# Dietary Fat Intake and Risk of Colorectal Cancer: A Systematic Review and Meta-Analysis of Prospective Studies

**DOI:** 10.3390/nu10121963

**Published:** 2018-12-12

**Authors:** Minkyeong Kim, Kyong Park

**Affiliations:** Department of Food and Nutrition, Yeungnam University, Gyeongsan, Gyeongbuk 38541, Korea; minkyeong@ynu.ac.kr

**Keywords:** dietary fats, fatty acids, colorectal neoplasms, systematic review, meta-analysis

## Abstract

Dietary fat intake is associated with the risk of colorectal cancer (CRC); however, the results of epidemiological studies on this are controversial. Therefore, this study aimed to summarize the available scientific evidence regarding the association between dietary fat and the risk of CRC. We conducted a systematic search of PubMed, Web of Science, and the Cochrane library for articles related to dietary fat and the risk of CRC. The summary relative risks with 95% confidence intervals (CI) were calculated via a random effect model. Begg’s test was used to detect publication bias. A total of 18 articles were identified. The pooled relative risk with 95% CI for the risk of CRC were 1.00 (95% CI: 0.90–1.12), 0.97 (95% CI: 0.86–1.10), 1.08 (95% CI: 0.92–1.26), and 0.99 (95% CI: 0.93–1.04) for total fat, saturated fatty acid, monounsaturated fatty acid, and polyunsaturated fatty acid, respectively. No significant associations were found in subgroup analyses. Begg’s test for all exposures revealed no publication bias (total fat, *p* = 0.3; saturated fatty acid, *p* = 0.1; monounsaturated fatty acid, *p* = 0.08; polyunsaturated fatty acid, *p* = 0.2). The studies included in this review and meta-analysis revealed that dietary fats and fatty acids had no effects on the risk of CRC.

## 1. Introduction

Colorectal cancer (CRC) is one of the most common cancers in both men and women worldwide [1,2,3]. The National Cancer Institute in the United States reported that mortality caused by CRC is the fourth highest among all cancers, accounting for 8.3% of all cancer-related deaths [4]. Moreover, the prevalence of CRC, which is common in Western countries, has been increasing rapidly in Asian countries, including South Korea, in recent decades [5,6]. In 1996, the national cancer screening program led by the National Health Insurance Corporation began to screen the five most prevalent cancers, including CRC, in South Korea [7]. Typically, colonoscopy is used as an initial screening tool for colorectal cancer, and a 10-year screening interval is recommended after initial negative colonoscopy [8]. However, recent studies have suggested the occurrence of “interval cancers” that involve polyps and cancers found some years after an initial negative colonoscopy; in a recent pooled analysis, the median time interval between the initial negative colonoscopy and colorectal cancer diagnosis among interval cancer cases was approximately 2.0–3.5 years [9].

Among factors associated with the development of CRC, modifiable risk factors, such as physical activity, smoking, alcohol drinking, and diet, have drawn attention [10,11]. In particular, fat intake is associated with the incidence of CRC [12,13,14,15]. Previous studies showed that CRC risk is higher in high fat-intake groups than low fat-intake groups [13,15,16]. Moreover, investigations into the risk of CRC and its association with different fat types revealed a higher CRC risk among those with high intake of both saturated fat and cholesterol (highest vs. lowest) [13]. A study involving Korean patients also reported that high intakes of saturated fatty acids (SFA) and monounsaturated fatty acids (MUFA) could increase the risk of CRC [15]. However, some studies found no associations of total fat or fatty acids with the risk of CRC [17,18,19,20,21,22,23]. Despite the controversy regarding the association between fat intake and CRC, no pooled analyses [24] have been performed since 2011; only individual studies have been conducted [17,25,26,27,28,29]. 

Accordingly, the need for meta-analyses that qualitatively assess individual studies to integrate recent literature on the association between total fat and individual fatty acid intake and the risk of CRC is evident. Therefore, in this study, we reviewed the available literature and summarized the results by conducting a meta-analysis of prospective cohort studies.

## 2. Materials and Methods 

### 2.1. Search Strategy

A systematic literature review and meta-analysis of studies on the association between dietary fat intake and the risk of CRC were conducted in accordance with the Preferred Reporting Items for Systematic Reviews and Meta-Analyses (PRISMA) guideline [30]. PubMed, Web of Science, and the Cochrane library electronic databases were searched to retrieve articles related to dietary fats/fatty acids and the risk of CRC published from January 1990 to June 2018. The following keywords were used: (colorectal OR colon OR rectal OR rectum OR large bowel) and (cancer OR carcinoma OR neoplasm) and (diet OR dietary OR fat OR fatty acid). The articles retrieved from the three databases were imported into the reference manager program, ENDNOTE X7 (Thomson Reuters, San Francisco, CA, USA). No language restrictions were imposed during the search. Additionally, the reference lists of included studies were manually retrieved to identify those that did not appear in the electronic databases but could be included in this meta-analysis. 

### 2.2. Study Selection 

Duplicates were removed using the ENDNOTE program. Titles and abstracts were reviewed to check whether the selected articles satisfied the inclusion criteria. If the eligibility for inclusion could not be determined based on the title and abstract only, the full text was reviewed for the final inclusion. Two investigators performed the selection process independently, and differences in opinion between investigators regarding study selection were resolved by discussion.

The inclusion criteria were as follows: (1) Original research, (2) prospective cohort study, (3) dietary fat as the major exposure factor (total fat, SFA, MUFA, and polyunsaturated fat or fatty acid (PUFA)), (4) CRC as the major outcome (colorectal cancer, colon cancer, rectal cancer, or large bowel cancer), and (5) relative risk (RR) or hazard ratio (HR) and the 95% confidence interval (CI) were reported. 

The exclusion criteria were as follows: (1) Papers other than an original research (reviews, letters, and comments, among others) and (2) not conducted in humans (animal, cell, in vivo, and in vitro, among others). For studies overlapping in studied populations, the most recently published article was included. 

### 2.3. Data Extraction and Quality Assessment

The following information was extracted by reviewing the full texts of the selected articles: Name of authors, year of publication, country, study population, sex, age at baseline, total number of participants, follow-up period, method of diet assessment, diagnostic criteria of CRC, intake levels of fat or fatty acids, RR/HR and 95% CI, and confounders. Quality evaluation was performed according to the Newcastle-Ottawa quality assessment scale-cohort studies [31], which comprises eight categories. A study earned 1 point for every item presents that was marked by a star sign, and 0 points if not. For the comparability category, 2 points were given if an item marked by 2 stars was mentioned in the article. Scores can range from 0 to 9 points, and only articles with scores of 6–9 points were included in the analysis. 

### 2.4. Statistical Analysis

To combine the effect size between dietary fat intake and the risk of CRC from each study, the RR or HR and 95% CI of the group with the highest fat intake relative to those of the group with the lowest fat intake were used. The weighted values needed to combine the effect sizes were calculated by identifying the standard deviation of a log-transformed RR, and the summary RR was determined using a random effect model. Cochrane’s Q and Higgin’s I^2^ tests were used to evaluate the heterogeneity of studies included in this analysis. Studies were deemed heterogeneous if I^2^ was >50% or the *p* value in the Cochrane’s Q test was <0.1 [32]. Subgroup analysis was conducted to identify the cause of heterogeneity and minimize it, and sensitivity analysis using the leave-one-out method was conducted. Begg’s and Mazumdar’s rank correlation test and a funnel plot test were used to detect publication bias that could affect the results of the meta-analysis. This meta-analysis was performed using Stata 14.0 (Stata Corp., College Station, TX, USA), and statistical significance was set at *p* <0.05.

## 3. Results

### 3.1. Literature Search

**Figure 1:**
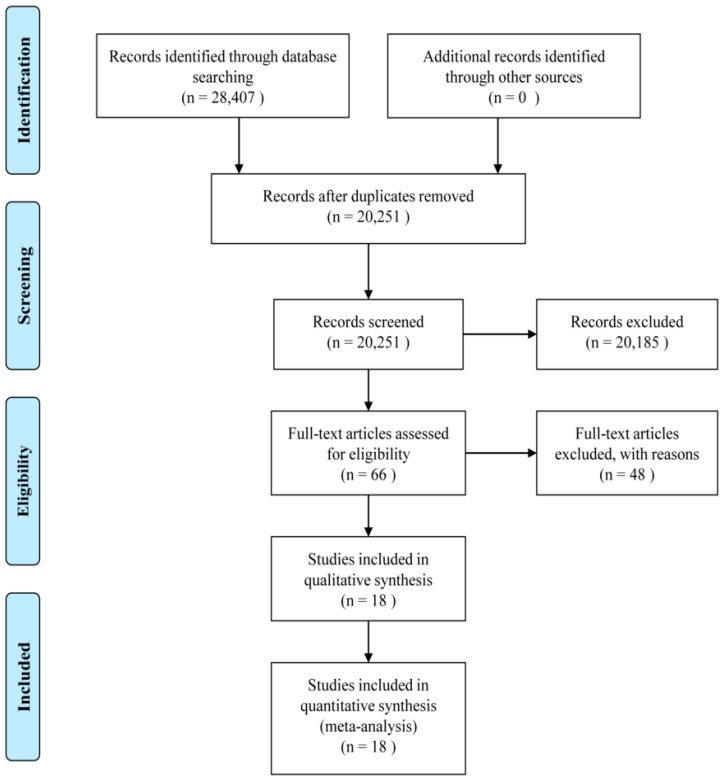
Preferred Reporting Items for Systematic Reviews and Meta-Analyses (PRISMA) diagram for study selection.

A total of 28,407 articles were identified in PubMed, Web of Science, and the Cochrane library. Duplicates (*n* = 8156), articles lacking information (*n* = 44), articles which were not original studies (*n* = 4065), articles not involving human participants (*n* = 8787), and articles unrelated to the topic of this study (*n* = 7288) were excluded. Among the 66 remaining studies, those with overlapping datasets (*n* = 6), those without RR or HR and 95% CI (*n* = 3), and those that were not prospective cohort studies (*n* = 39) were excluded. Overall, 18 articles were included in this meta-analysis (Figure 1).

### 3.2. Study Characteristics and Quality Assessment

Table S1 summarizes the characteristics of the 18 studies included in this study. Of these, 11, 9, 7, and 14 studies analyzed the total fat [17,18,19,25,33,34,35,36,37,38,39], SFA [18,19,29,33,35,36,37,38,39], MUFA [18,19,33,34,36,37,39], and PUFA (total PUFA and omega-3 and omega-6 fatty acids [17,18,19,26,27,28,29,33,36,37,39,40,41,42]) as exposure factors, respectively. Ten studies were conducted in the United States, four in Europe, and four in Asia. 

Table S2 summarizes the results of the quality assessment using the Newcastle-Ottawa quality assessment scale-cohort studies. The 18 studies had a mean quality score of 7.4 points. 

### 3.3. Dietary Fat Intake and Incidence of Colorectal Cancer

**Figure 2:**
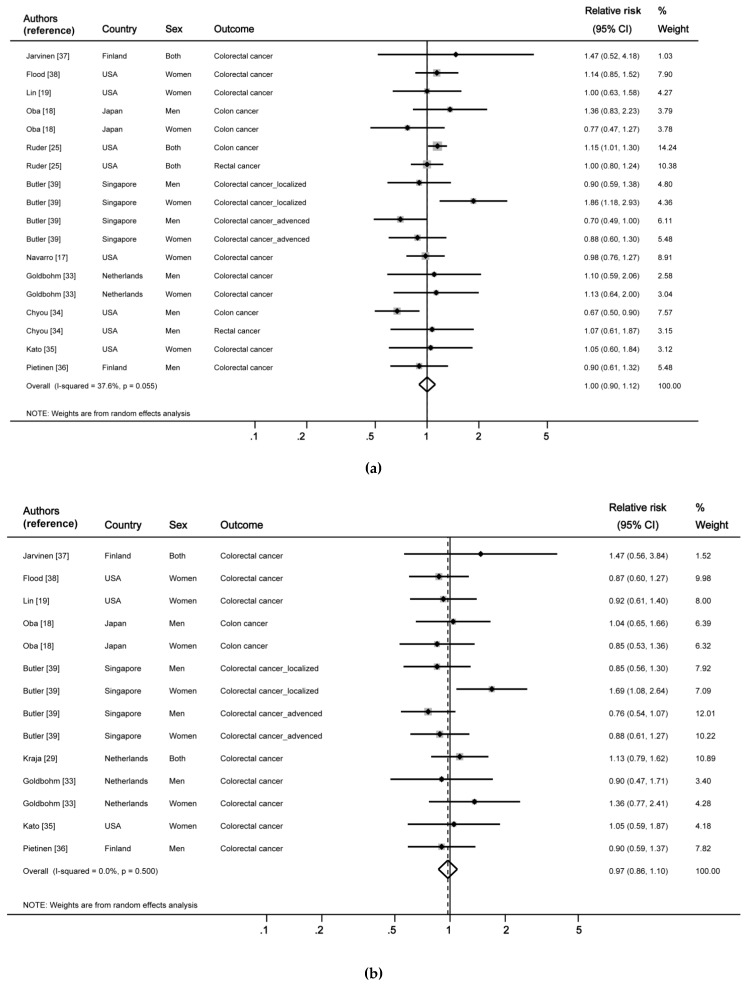

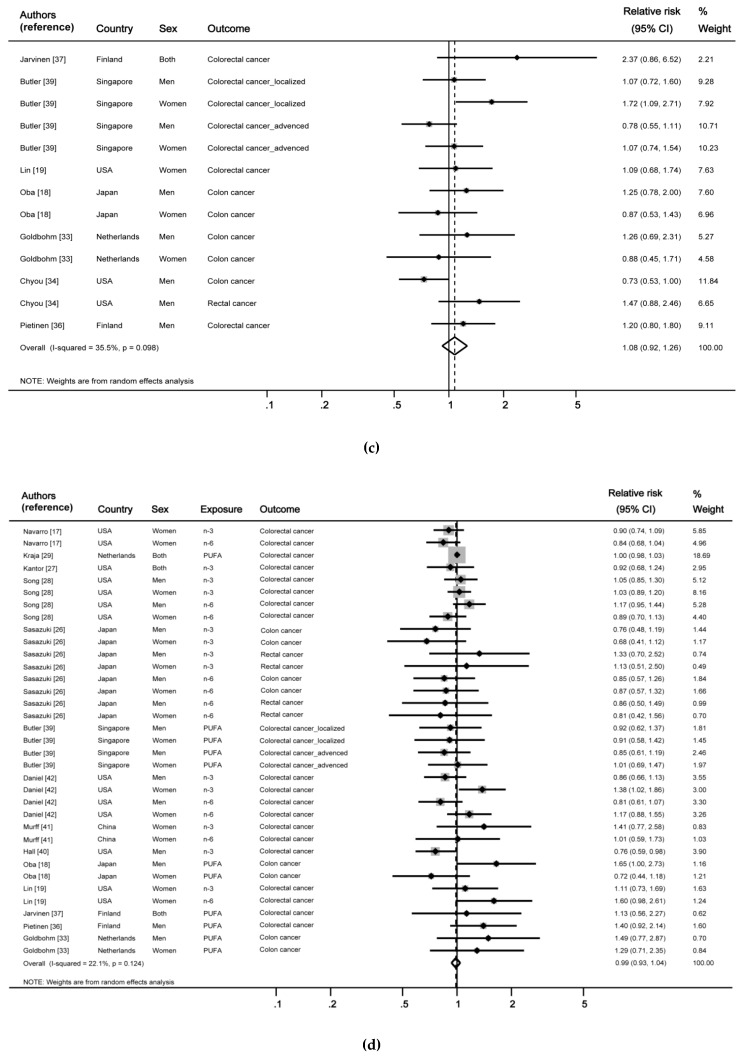
Forest plot of pooled estimates of the association between dietary fat and fatty acid intake and risk of colorectal cancer. (**a**) Total fat intake and risk of colorectal cancer; (**b**) saturated fat or fatty acid and colorectal cancer; (**c**) monounsaturated fat or fatty acid and colorectal cancer; and (**d**) polyunsaturated fat or fatty acid and colorectal cancer. CI, confidence interval; n-3, omega-3 fatty acids; n-6, omega-6 fatty acids.

Figure 2 shows the results integrating the analytical studies on the association between total fat and fatty acids and the risk of CRC. When the results of 11 articles (18 studies reporting the investigated association between total fat intake and CRC) were summarized, no significant association was observed (RR: 1.00, 95% CI: 0.90–1.12). In addition, no heterogeneity was observed in the nine articles reporting the association between SFA and the risk of CRC when the results were combined (I^2^ = 0.0%, *p* = 0.5), and no significant association was also observed (RR: 0.97, 95% CI: 0.86–1.10). Similarly, the pooled results of the seven articles reporting the MUFA-CRC association were not significant (RR: 1.08, 95% CI: 0.92–1.26). The results of the 14 articles on PUFA, including omega-3 and omega-6 fatty acids, were pooled, and no significant association between PUFA intake and risk of CRC was found (RR: 0.99, 95% CI: 0.93–1.04).

In the subgroup analysis of the association between total fat intake and CRC according to the continent in which the study was conducted, heterogeneity was reduced in the United States and Europe, but the association was not significant (Table 1). Similarly, no significant associations were observed for other types of fat, including SFA, MUFA, and PUFA, in the subgroup analysis by continent. Furthermore, no significant differences were observed for all exposure factors in the subgroup analyses either by sex or the follow-up period, and no association was observed between total fat intake and CRC in the subgroup analysis according to the type of CRC.

To verify the reliability of these results, we conducted the leave-one-out analysis to assess the influence of each study on the summary RR. When the study by Chyou et al., (including men) was excluded [34], the association between total fat intake and CRC showed a positive direction, but the statistical significance did not change. When the study by Butler et al., (including women) was removed [39], the RR of CRC in association with SFA intake showed a negative direction, but the statistical significance did not change. Excluding any of the other studies showed results similar to those of the integrated results. The results for MUFA and PUFA were also similar to the summary RR when the leave-one-out analysis was performed.

### 3.4. Publication Bias

Publication bias was visually analyzed using the funnel plot, and the test statistics were determined using Begg’s and Mazumdar’s rank correlation test. The funnel plot for total fat intake showed an asymmetrical trend, but Begg’s test showed no publication bias (*p* = 0.3). Moreover, SFA showed an asymmetrical trend at the lower right side of the funnel plot; however, Begg’s test also showed no publication bias (*p* = 0.1). Similarly, no publication bias was observed on the funnel plots, and Begg’s test results for MUFA and PUFA also showed no publication bias (Begg’s test = 0.08 and 0.2, respectively). 

## 4. Discussion

This study evaluated the influence of dietary fat and fatty acids on the incidence of CRC using a systematic review and meta-analysis. The results showed that intakes of total fat, SFA, MUFA, and PUFA were not associated with the risk of CRC, and no differences were observed in the subgroup analyses by sex, continent, follow-up period, or CRC type.

Animal model studies conducted on dietary fat intake (approximately 35%–45% of the total energy intake) and risk of CRC showed a positive association [16,43]. The mechanism used to explain this association was the increased production of secondary bile acids by gut microbes by a high-fat diet, which subsequently increases pro-inflammatory effectors, and promotes the development of CRC by inducing oxidative stress [44]. A cohort study published in 1990 reported that high intakes of total fat, animal fat, and MUFA increased the risk of colon cancer [45]. Nevertheless, most recent epidemiological studies showed no significant association between fat intake and the risk of CRC [17,18,19,33,38], with inconsistent health effects of fats and fatty acids based on the results of animal model studies. Independent health effects of dietary fats on CRC were controversial; instead, the high intake of red and processed meat (which are animal sources of fat), has been constantly reported to be associated with the risk of CRC [46,47,48].

In our study, we found that PUFA intake was not associated with CRC. Some studies have reported a positive association between levels of PUFA intake and CRC [49,50], whereas numerous epidemiologic studies have reported no association [18,29,33,36,37,39]. Unlike other types of fat, higher levels of omega-3 fatty acids, which are known to reduce inflammatory reactions [44], showed a significant association with the decreased risk of CRC in some studies [40,42], but not in most previous studies [26,27,28,29,39]. Biological mechanisms have been used to explain the effect of dietary fat intake on the development of colon cancer through previous animal model studies; however, significant associations were not shown in most human studies. Until now, epidemiological studies have not shown clear evidence of an association between dietary fat intake and the risk of CRC.

Although multivariable-adjusted RRs were reported in individual studies, the range of adjustment varied across studies, with the possibility of residual confounding. However, the possibility of reverse causality or information bias was minimized by integrating results of the prospective cohort study. Moreover, unlike previous studies, the current study assessed the quality of articles included in the meta-analysis to prevent the distortion of research results by poorly designed studies or from small study effects. Finally, because no meta-analyses integrated the research results on this topic since 2011, this study included studies published after 2011 to enhance the evidence level on the unclear association between dietary fat intake and CRC. 

## 5. Conclusions

This study systematically reviewed research articles published since the 1990s and integrated the results to assess the influence of dietary fat or fatty acids on the risk of CRC. No significant association was observed between total fat, SFA, MUFA, and PUFA intakes and the risk of CRC. Moreover, subgroup analyses by sex, continent, and follow-up period revealed no significant differences. Because numerous studies have been published in the United States and Europe, large-scale prospective studies and clinical trials involving a greater diversity of countries and races should be conducted.

## Figures and Tables

**Table 1:** Pooled estimates for the association between dietary fat/fatty acids and colorectal cancer risk by subgroups.

Indexes	No. of Studies	RR (95% CI)	Heterogeneity	
			I^2^	*p* value
**Total Fat**	18			
**Continent**				
American and European	12	1.02 (0.93–1.13)	12.3	0.3
Asian	6	1.00 (0.74–1.34)	64.6	0.02
**Sex**				
Men	7	0.87 (0.72–1.05)	29.1	0.2
Women	8	1.06 (0.90–1.25)	23.2	0.2
**Follow-up Period**				
<9 years	8	1.05 (0.89–1.22)	0.0	0.8
≥9 years	10	0.98 (0.83–1.15)	61.8	0.005
**Cancer Type**				
Colorectal Cancer	12	1.01 (0.88–1.16)	19.6	0.3
Colon Cancer	4	0.95 (0.68–1.32)	77.2	0.004
Rectal Cancer	2	1.01 (0.82–1.23)	0.0	0.8
**Saturated Fat/Fatty Acid**	14			
**Continent**				
American and European	8	1.00 (0.85–1.19)	0.0	0.9
Asian	6	0.96 (0.76–1.20)	43.5	0.1
**Sex**				
Men	5	0.86 (0.71–1.05)	0.0	0.9
Women	7	1.03 (0.84–1.25)	25.3	0.2
**Follow-up Period**				
<9 years	8	0.95 (0.81–1.12)	0.0	0.9
≥9 years	6	1.02 (0.80–1.31)	49.5	0.1
**Monounsaturated Fat/Fatty Acid**	13			
**Continent**				
American and European	22	1.09 (0.85–1.41)	41.1	0.1
Asian	6	1.07 (0.86–1.34)	40.4	0.1
**Sex**				
Men	7	0.99 (0.85–1.15)	40.8	0.1
Women	5	1.12 (0.88–1.42)	20.0	0.3
**Follow-up Period**				
<9 years	6	1.10 (0.90–1.35)	0.0	0.9
≥9 years	7	0.10 (0.84–1.44)	63.2	0.01
**Polyunsaturated Fat/Fatty Acid ^1^**	35			
**Continent**				
American and European	19	1.00 (0.94–1.07)	38.0	0.04
Asian	16	0.93 (0.82–1.04)	0.0	0.6
**Sex**				
Men	20	0.97 (0.86–1.10)	40.1	0.06
Women	23	0.99 (0.91–1.09)	19.5	0.2
**Follow-up Period**				
<9 years	12	1.11 (0.95–1.30)	48.4	0.03
≥9 years	23	0.99 (0.97–1.02)	0.0	0.6

Abbreviation: RR, relative risk; CI, confidence interval. ^1^ Polyunsaturated fat or fatty acid including omega-3 and omega-6 fatty acids.

## Supplementary Materials

The following are available online at http://www.mdpi.com/2072-6643/10/12/1963/s1, Table S1: Characteristics of studies included on dietary intakes of fat and fatty acids and the risk of colorectal cancer, Table S2: Assessment of quality using the Newcastle-Ottawa quality assessment scale-cohort studies.
