# Supplementary material for: Dietary Fat Intake and Risk of Colorectal Cancer: A Systematic Review and Meta-Analysis of Prospective Studies
Source: Nutrients. 2018 Dec 12;10(12):1963. doi: 10.3390/nu10121963 (PMC6315498; doi:10.3390/nu10121963)
Supplement: Supplementary file 1 [file nutrients-10-01963-s001.pdf]

Table S1. Characteristics of studies included on dietary intakes of fat and fatty acids and the risk of colorectal cancer.

Author (year)	Country	Sex	Age at baseline (year)	No. of participant	Follow-up	Dietary assessment	Methods of diagnosing CRC	Exposure	Exposure intake level	Outcome	RR (95% CI)	Confounding factor	Name of study
Goldbohm (1994)	Netherlands	B	55-69	120,852	3.3 y	150-item SQFFQ (validated)	Record linkage with all nine regional cancer registries in the Netherlands and with PALGA, a national data base of pathology reports	Fat (g/day)	Lowest: 76 Highest: 111	CC	M: 1.10 (0.59-2.07) W: 1.13 (0.64-2.00)	Sex and dietary fiber intake	The Netherlands Cohort Study
								SFA (g/day)	Lowest: 28 Highest: 47	CC	M: 0.90 (0.47-1.70) W: 1.36 (0.77-2.42)		
								MUFA (g/day)	Lowest: 27 Highest: 43	CC	M: 1.26 (0.69-2.31) W: 0.88 (0.45-1.69)		
								PUFA (g/day)	Lowest: 11 Highest: 31	CC	M: 1.49 (0.77-2.86) W: 1.29 (0.71-2.35)		
Chyou (1996)	USA	M	≥45	7,945	22 y (174,514 PY)	24-hour diet recall	Oahu hospitals and periodic linkage with the Hawaii Tumor Registry	Fat (kcal from fat, %)	Lowest: <27 Highest: ≥40	CC	0.67 (0.50-0.91)	Age	Honolulu Heart Program
										RC	1.07 (0.61-1.86)		
								MUFA (g/day)	Lowest: <22 Highest: ≥40	CC	0.73 (0.53-1.00)		
										RC	1.47 (0.88-2.47)		
Kato (1997)	USA	W	34-65	14,727	7.1 y	70-item SQFFQ	Medical records and pathological diagnosis	Total fat	-	CRC	1.05 (0.60-1.84)	Total calorie intake, age, place at enrollment, and highest level of education	The New York University Women's Health Study
								SFA		CRC	1.05 (0.59-1.88)		
Pietinen (1999)	Finland	M	50-69	27,111	8 y	276-item SQFFQ (validated)	Finnish Cancer Registry, two oncologists and a pathologist checked the original histological specimens	Total fat (g)	Lowest: 81.6 Highest: 119.0	CRC	0.90 (0.60-1.30)	Age (5-year categories), use of supplement, smoking years, body mass index, alcohol, education, physical activity at work, and calcium intake	Alpha-Tocopherol, Beta-Carotene Cancer Prevention Study
								SFA (g)	Lowest: 33.8 Highest: 65.1	CRC	0.90 (0.60-1.40)		
								MUFA (g)	Lowest: 28.4 Highest: 40.7	CRC	1.20 (0.80-1.80)		
								PUFA (g)	Lowest: 6.5 Highest: 19.4	CRC	1.40 (0.90-2.10)		
								n-3 fish fatty acid (g)	Lowest: 0.2 Highest: 0.7	CRC	1.20 (0.80-1.90)		
Jarvinen (2001)	Finland	B	Case: 49.5 Control: 39.0 (adjusted for sex)	9,959	32 y	Interview (100-item questionnaire)	The national Finnish Cancer Registry	Total fat (g)	M Lowest: <95.7 Highest: >151.5	CRC	1.47 (0.52–4.20)	Age, sex, body mass index, occupation, smoking, geographical area, energy intake and consumption of vegetables, fruits and cereals	The Finnish Mobile Clinic Health Examination Survey

									W<br>Lowest: <64.7<br>Highest: >105.5				
								SFA (g)	M<br>Lowest: <53.5<br>Highest: >86.6<br><br>W<br>Lowest: <35.6<br>Highest: >60.1	CRC	1.47 (0.56–3.83)		
								MUFA (g)	M<br>Lowest: <30.5<br>Highest: >49.2<br><br>W<br>Lowest: <20.8<br>Highest: >34.0	CRC	2.37 (0.86–6.51)		
								PUFA (g)	M<br>Lowest: <5.9<br>Highest: >10.3<br><br>W<br>Lowest: <4.1<br>Highest: >7.5	CRC	1.13 (0.56–2.26)		
Flood (2003)	USA	W	61.9	45,496	8.5 y (386,716 PY)	62-item FFQ (validated)	Self-reports, pathology reports, International Classification of Diseases for Oncology	Total fat (% of energy)	Lowest: 23.9<br>Highest: 45.3	CRC	1.14 (0.86-1.53)	Energy using the multivariate nutrient density method, controlling for total fat (only saturated and unsaturated fat)	The Breast Cancer Detection Demonstration Project
								SFA (% of energy)	Lowest: 7.1<br>Highest: 15.7	CRC	0.87 (0.60-1.27)		
Lin (2004)	USA	W	≥45	37,547	8.7 y	131-item FFQ (validated)	Medical records and pathology reports	Total fat (% energy)	Lowest: 22<br>Highest: 38	CRC	1.00 (0.63-1.58)	Age, random treatment assignment, body mass index, family history of colorectal cancer, history of colorectal polyps, physical activity, cigarette smoking, alcohol consumption, postmenopausal hormone therapy, and total energy intake	The Women's Health Study
								SFA (% energy)	Lowest: 7<br>Highest: 13	CRC	0.92 (0.61-1.41)		
								MUFA (% energy)	Lowest: 8<br>Highest: 15	CRC	1.09 (0.68-1.73)		
								n-3 PUFA (% energy)	Lowest: 0.03<br>Highest: 0.21	CRC	1.11 (0.73-1.69)		
								n-6 PUFA (% energy)	Lowest: 3.8<br>Highest: 7.6	CRC	1.60 (0.98-2.60)		
Oba (2006)	Japan	B	M: 54.5<br>W: 55.7	31,551	8 y	SQFFQ (validated)	Medical records	Total fat (g)	Lowest: 40.9	CC	M: 1.36 (0.83–	Age, height, body mass index, total pack-years of cigarette smoking, alcohol intake, and physical activity	A community-based cohort in Japan

									Highest: 67.6		2.24) W: 0.77 (0.47–1.27)		
								SFA (g)	Lowest: 10.9 Highest: 19.5	CC	M: 1.04 (0.65–1.66) W: 0.85 (0.53–1.36)		
								MUFA (g)	Lowest: 13.7 Highest: 23.9	CC	M: 1.25 (0.78–1.99) W: 0.87 (0.53–1.44)		
								PUFA (g)	Lowest: 11.1 Highest: 18.9	CC	M: 1.65 (1.00–2.74) W: 0.72 (0.44–1.18)		
								Long n-3 fatty acids (mg)	Lowest: 462 Highest: 1405	CC	M: 1.24 (0.80–1.95) W: 0.89 (0.56–1.44)		
Hall (2008)	USA	M	53.4-54.0	21,406	22 y	SQFFQ (validated)	Medical records and pathology report	n-3 fatty acid from fish	-	CRC	0.76 (0.59-0.98)	Age, smoking, body mass index, multivitamin use, history of diabetes, use of aspirin, exercise, red meat intake	The Physicians' Health Study
Butler (2009)	Singapore	B	M: 54-57 W: 53-58	61,321	9.8 y	165-item QFFQ (validated)	Record linkage of the cohort database	Total fat (g/1000kcal)	M Lowest: 18.3 Highest: 31.5 W Lowest: 18.9 Highest: 31.7	CRC (localized)	M: 0.90 (0.59–1.38) W: 1.86 (1.18–2.92)	Age at interview, use of dialect, interview year, diabetes at baseline, smoking history, body mass index, alcohol intake, education, physical activity, first degree relative diagnosed with colorectal cancer, and total daily energy intake	The Singapore Chinese Health Study
										CRC (advanced)	M: 0.70 (0.49–1.00) W: 0.88 (0.60–1.30)		
								SFA (g/1000kcal)	M Lowest: 5.9 Highest: 11.8 W Lowest: 6.0 Highest: 11.9	CRC (localized)	M: 0.85 (0.56–1.30) W: 1.69 (1.08–2.63)		
										CRC (advanced)	M: 0.76 (0.54–1.07) W: 0.88 (0.61–1.28)		
								MUFA (g/1000kcal)	M Lowest: 6.0 Highest: 10.9 W Lowest: 6.2 Highest: 10.9	CRC (localized)	M: 1.07 (0.72–1.60) W: 1.72 (1.09–2.70)		
										CRC (advanced)	M: 0.78 (0.55–1.11) W: 1.07 (0.74–1.54)		

								Total PUFA (g/1000kcal)	M Lowest: 3.2 Highest: 7.2	CRC (localized)	M: 0.97 (0.65–1.44) W: 0.91 (0.58–1.43)		
									W Lowest: 3.3 Highest: 7.4	CRC (advanced)	M: 0.86 (0.61–1.20) W: 1.03 (0.70–1.51)		
								Total n-3 PUFA (g/1000kcal)	M Lowest: 0.35 Highest: 0.66	CRC (localized)	M: 0.78 (0.51–1.21) W: 1.19 (0.75–1.89)		
									W Lowest: 0.36 Highest: 0.67	CRC (advanced)	M: 1.09 (0.80–1.50) W: 1.09 (0.75–1.59)		
								n-6 PUFA (g/1000kcal)	M Lowest: 2.8 Highest: 6.5	CRC (localized)	M: 0.92 (0.62–1.37) W: 0.91 (0.58–1.42)		
									W Lowest: 2.9 Highest: 6.7	CRC (advanced)	M: 0.85 (0.61–1.19) W: 1.01 (0.69–1.47)		
Daniel (2009)	USA	B	M: 70.0-70.5 W: 68.0-69.0	99,080	6 y	152-item FFQ (validated)	Self-report, medical records, International Classification of Diseases for Oncology	Total n-3 (g/d)	Lowest: <0.93 Highest: ≥1.38	CRC	M: 0.86 (0.66-1.13) W: 1.38 (1.02-1.85)	Age, energy, hormone replacement therapy (in women only), recreational physical activity, NSAID use, colorectal screening, body mass index, and red and processed meat, low-fat dairy, fruit, and vegetable intake	The Cancer Prevention Study-II Nutrition Cohort
								Total n-6 (g/d)	Lowest: <8.4 Highest: ≥12.1	CRC	M: 0.81 (0.61-1.07) W: 1.17 (0.88-1.55)		
Murff (2009)	China	W	40-70	73,242	11 y	FFQ	Biennial in-home interviews and annual record linkage	Total n-3 (g/d)	Lowest: 0.64 Highest: 1.61	CRC	1.41 (0.77-2.57)	Age, energy intake, total energy-adjusted n-3 PUFA intake (total n-3), total energy-adjusted n-6 PUFA intake (total n-6), energy-adjusted ratio of total n-6 PUFA to n-3 PUFA intake (only total n-3 and n-6), body mass index, current smoker, alcohol use, regular physical activity in past 5 y, total energy-adjusted red meat intake, menopausal status, hormone replacement therapy use, multivitamin use, and aspirin use. PUFA is only adjusted for ratio n-6/n-3	The Shanghai Women's Health Study
								Total n-6 (g/d)	Lowest: 4.28 Highest: 9.56	CRC	1.01 (0.59-1.73)		
Ruder (2011)	USA	B	50-71	292,797	11 y	37-item FFQ	Probabilistic linkage with state cancer registries	Total fat (g/1000 kcal)	Lowest: 35 Highest: 52	CC	1.15 (1.01–1.30)	Energy at ages 12–13 years, energy in recent adulthood, nutrient of interest in recent adulthood, age at completion of risk-factor questionnaire, sex, body mass index, race, education, physical activity, alcohol consumption, smoking, use of nonsteroidal anti-inflammatory drugs, use of hormone replacement therapy, self-report of a first-degree relative with a history of colon cancer, and recent adult intake	The NIH-AARP Diet and Health Study
									Lowest: 35 Highest: 52	RC	1.00 (0.81–1.25)		
Sasazuki (2011)	Japan	B	40–69	121,021	9.3 y (827,833 PY)	138-item FFQ (validated)	Major local hospitals in the study area and from data linkage with population-based cancer	Total n-3 (g/d)	M Lowest: 1.79	CC	M: 0.76 (0.48–1.18) W: 0.68	Age, area, body mass index, smoking status, alcohol drinking, past history of or medication use for diabetes mellitus, METs, screening for CRC, total calorie, calcium, vitamin D, fiber and red meat intake	Japan Public Health Center–based prospective study

							registries, International Classification of Diseases for Oncology		Highest: 4.48		(0.41–1.12)		
									W Lowest: 2.13 Highest: 4.48	RC	M: 1.33 (0.70–2.51) W: 1.13 (0.51–2.49)		
								Total n-6 (g/d)	M Lowest: 5.85 Highest: 11.97	CC	M: 0.85 (0.57–1.25) W: 0.87 (0.57–1.31)		
									W Lowest: 6.56 Highest: 11.72	RC	M: 0.86 (0.50–1.50) W: 0.81 (0.42–1.56)		
Kantor (2014)	USA	B	50-76	68,109	6.7 y	120-item FFQ	Linkage to the western Washington SEER registry, pathologists to identify cases	Dietary EPA+DHA (g/d)	Lowest: <0.08 Highest: ≥0.29	CRC	0.92 (0.68-1.24)	Age, sex, race/ethnicity, education, body mass index, energy intake, MET-hours per week of moderate/vigorous activity, alcohol intake, smoking history, multivitamin use, calcium intake, dietary fiber intake, fruit and vegetable intake, red/processed meat intake, aspirin use, non-aspirin NSAID use, family history of colorectal cancer, history of sigmoidoscopy/colonoscopy, history of polyps, hormone replacement therapy, cardiovascular disease, memory loss, use of cholesterol-lowering drugs, and omega-6 (linoleic +arachidonic) intake	VITamins And Lifestyle
Song (2014)	USA	B	M: 40-75 W: 30-55	M: 47,143 W: 76,386	M: 23 y W: 21 y	116- and 131-item FFQs (validated)	Biennial questionnaires, medical records and pathology reports	Marine n-3 (g/d)	Lowest: <0.16 Highest: ≥0.41	CRC	M: 1.05 (0.85–1.30) W: 1.03 (0.89–1.20)	Age, calendar year, family history of colorectal cancer, prior lower gastrointestinal endoscopy, pack-years of smoking before age 30, body mass index, physical activity, current multivitamin use, postmenopausal status and hormone use, regular aspirin or NSAID use, total caloric intake, red meat, process meat, alcohol consumption and energy-adjusted intake of folate, calcium, vitamin D and total fiber	The NHS and HPFS cohorts
								Total n-6 (g/d)	Lowest: <10.0 Highest: ≥14.0	CRC	M: 1.17 (0.95–1.44) W: 0.89 (0.70–1.12)		
Kraja (2015)	Netherlands	B	≥55	4,967	14.6 y (72,526 PY)	170-item SQFFQ (validated)	Physicians assessed pathology data and medical records, International Classification of Disease	Total PUFA (g/d)	Lowest: 8.7 Highest: 21.7	CRC	0.95 (0.60-1.32)	Age, gender, energy-adjusted dietary fiber intake, and Dutch Healthy Diet index (excluding PUFA, fish, SFA, and dietary fiber components)	The Rotterdam Study
								n-6 PUFA (g/d)	Lowest: 6.1 Highest: 18.6	CRC	0.89 (0.65-1.23)		
								n-3 PUFA (g/d)	Lowest: 0.7 Highest: 1.5	CRC	1.44 (1.02-2.04)		
								SFA (g/d)	Lowest: 25.2 Highest: 38.5	CRC	1.13 (0.79-1.62)		
Navarro (2016)	USA	W	Case: 66 Non-case: 63	134,017	11.7 y	122-item FFQ	Pathology reports and medical records	Total fat (g/day)	Lowest: <33.1 Highest: >80.6	CRC	0.98 (0.76-1.27)	Total energy intake, age, body mass index, education, family history of colorectal cancer, history of colonoscopy, current NSAID use, alcohol intake, smoking history, physical activity, ever use of hormone therapy, folate, calcium, and red meat intake, study component and CT randomization assignment and treatment arm	The Women's Health Initiative prospective cohort
								n-6 PUFA (g/day)	Lowest: <5.9 Highest: >14.6	CRC	0.84 (0.68-1.05)		
								n-3 PUFA (g/day)	Lowest: <0.80	CRC	0.90 (0.74-1.09)		

									Highest: >1.90				

Abbreviation: CRC, colorectal cancer; CC, colon cancer; RC, rectal cancer; RR, relative risk; CI, confidence interval; B, both (men and women); y, year; SQFFQ, semi-quantitative food frequency questionnaire; PALGA, the nationwide network and registry of histo- and cytopathology in the Netherlands; SFA, saturated fat/fatty acid; MUFA, monounsaturated fat/fatty acid; PUFA, polyunsaturated fat/fatty acid; USA, United States of America; M, men; PY, person-year; W, women; FFQ, food frequency questionnaire; QFFQ, quantitative food frequency questionnaire; NSAID, non-steroidal anti-inflammatory drugs; NIH-AARP, National Institute of Health American Association of Retired Persons; MET, metabolic equivalent; SEER, Surveillance, Epidemiology, and End Results; EPA, eicosapentaenoic acid; DHA, docosahexaenoic acid; NHS, Nurses' Health Study; HPFS, Health Professionals Follow-Up Study; CT, clinical trial.

Table S2. Assessment of quality using the Newcastle-Ottawa quality assessment scale-cohort studies.

Author (year)	Selection				Comparability	Outcome			Total score
	1) Representativeness of the exposed cohort	2) Selection of the non-exposed cohort	3) Ascertainment of exposure	4) Demonstration that outcome of interest was not present at start of study	1) Comparability of cohorts on the basis of the design or analysis	1) Assessment of outcome	2) Was follow-up long enough for outcomes to occur	3) Adequacy of follow up of cohorts	
**Goldbohm (1994)**	★	★	★	★	★	★	-	★	**7**
**Chyou (1996)**	-	★	★	★	★	★	★	★	**7**
**Kato (1997)**	★	★	-	★	★	★	★	★	**7**
**Pietinen (1999)**	-	★	★	★	★★	★	★	-	**7**
**Jarvinen (2001)**	★	★	★	★	★★	★	★	-	**8**
**Flood (2003)**	★	★	★	★	★	-	★	★	**7**
**Lin (2004)**	-	★	★	★	★★	★	★	-	**7**
**Oba (2006)**	★	★	★	★	★	★	★	-	**7**
**Hall (2008)**	-	★	★	★	★	★	★	★	**7**
**Butler (2009)**	-	★	★	★	★★	★	★	★	**8**
**Daniel (2009)**	★	★	★	★	★★	★	★	★	**9**
**Murff (2009)**	★	★	★	★	★★	★	-	★	**8**
**Sasazuki (2011)**	★	★	★	★	★★	★	★	★	**9**
**Ruder (2011)**	★	★	-	★	★★	★	-	★	**7**
**Song (2014)**	-	★	★	★	★★	★	★	★	**8**
**Kantor (2014)**	★	★	-	★	★★	★	★	-	**7**
**Kraja (2015)**	★	★	★	★	★	★	★	-	**7**
**Navarro (2016)**	-	★	-	★	★★	★	★	-	**6**
